# Lightweight and robust image steganography method for secure communication

**DOI:** 10.1371/journal.pone.0357380

**Published:** 2026-09-16

**Authors:** Wanjie Kang, Youshun Pan

**Affiliations:** School of Automation Engineering, Moutai Institute, Renhuai, China; Department of Technical Education, Training and Skill Development, West Bengal, INDIA

## Abstract

Image steganography plays a crucial role in covert communication and copyright protection, but existing methods still struggle to achieve a balance between embedding capacity, image imperceptibility, anti-detection capabilities, and model complexity. Under complex channel interference such as compression and noise, the accuracy of secret information recovery easily declines, while computational costs increase. Therefore, this study proposes an image steganography method based on an end-to-end embedding-extraction network and lightweight convolutional modules. First, the carrier image and the secret image are input into the encoding network, where multi-scale spatial and channel features are extracted using improved Inception-V3 and Xception convolutional modules. Second, a RepVGG structure reparameterization mechanism is introduced to reduce computational complexity in the inference stage while maintaining feature representation capabilities. Finally, a dense connection mechanism is combined in the decoding network to enhance shallow feature reuse, and a discriminant network applies adversarial constraints to the carrier image and the steganographic image. Experimental results demonstrate that the proposed algorithm achieves a peak signal-to-noise ratio of 40.7 dB, a structural similarity index of 0.969, and a learned perceptual image patch similarity of 0.055 on the test set, all outperforming comparative methods. In terms of steganographic performance, its bit error rate is only 0.008, and the area under the curve is 0.398, also surpassing the other two comparative algorithms. Additionally, under strong compression conditions of 30, the proposed algorithm maintains a recovery accuracy of 0.82 and controls the bit error rate at 0.38, while under 90 compression, it achieves a recovery accuracy of 0.98 with the bit error rate dropping to 0.23. In noise interference experiments, the method retains a recovery accuracy of 0.96 and a bit error rate of 0.22 when the noise standard deviation is 0.05, demonstrating strong robustness. The findings indicate that this algorithm achieves a balance between performance and efficiency in steganographic capacity, image quality, and anti-interference capability, offering a more practical solution for image steganography applications.

## 1. Introduction

With the ongoing progress of information network, the security and privacy of data transmission become particularly important [1]. Image steganography is a kind of covert communication method. The image to be transmitted is embedded with encrypted information. Meanwhile, it ensures that the image does not reflect any abnormalities, and the receiver can accurately extract the hidden information [2]. The difference between steganography and traditional cryptography is that it emphasizes the imperceptibility of embedded information and can be more suitable for financial transactions, digital copyright protection and other fields [3]. With the continuous expansion of the volume of multimedia data, there are more and more network attacks, and privacy leaks occur frequently. How to ensure the security and concealment of embedded information while ensuring the convenience of steganography algorithm has become the focus of industry research.

Researchers proposed a substantial number of image steganography methods of various types. For instance, R Singh et al. proposed a medical image watermarking method based on Discrete Wavelet Transform (DWT) and Advanced Encryption Standard (AES). This scheme embeds patient data into encrypted medical images using two-level DWT, demonstrating excellent robustness while ensuring imperceptibility. The results validated the robustness and imperceptibility of the proposed scheme [4]. Y Huo et al. proposed a deep learning (DL)-based steganography method suitable for High Dynamic Range (HDR) images in the OpenEXR format. This marked the first application of DL to HDR image steganography and the realization of hidden image embedding. The method embedded Low Dynamic Range (LDR) secret images into the mantissa of HDR carrier images of the same size using a hiding network and recovered them at the receiving end through an extraction network. Experiments showed that this algorithm outperformed existing similar methods in regard to security, robustness, and embedding capacity [5]. To enhance the security of image steganography, A Alenizi et al. proposed an information hiding method based on minimizing image bit variance. This method incorporated an encryption mechanism to encrypt and verify data before embedding and combined hash table encryption to strengthen communication security, ensuring the covert transmission of text information within images. To further enhance protection, a secondary encryption-decryption process based on different hash algorithms was introduced to process the stego images [6]. P Naveen et al. proposed a steganography method that integrated the Least Significant Bit (LSB) technique with Genetic Algorithm (GA). This method enabled the embedding of multiple color images into a single monochrome image, thereby increasing the embedding capacity of secret data. Furthermore, the introduction of GA further enhanced the security of the transmission process. Experimental results indicated that the quality of the stego and extracted images was excellent, with Structural Similarity Index Measure (SSIM) values ranging from 0.996 to 1.000, achieving lossless and secure transmission of multicolor images [7].

To address the problems that traditional cryptography cannot hide the existence of communication and that steganography cannot recover the quality of the carrier without loss, R Anushiadevi et al. proposed a separable encrypted image reversible data hiding scheme. By combining histogram-free translation multi-bit reversible data hiding technology with additive homomorphic encryption, a high embedding capacity of 0.691 bpp was achieved [8]. To address the issues of insufficient imperceptibility and noise robustness of hidden images in image steganography, as well as the lack of interactive performance evaluation methods, M Chinnusami et al. proposed a hybrid image hiding method based on integer wavelet transform and singular value decomposition. By integrating integer wavelet transform and singular value decomposition techniques, the imperceptibility and robustness of the hidden image are improved [9]. C Lakshmi et al. proposed a three-layer feature-dependent watermarking scheme based on DWT, to solve the problem of image ownership authentication in digital e-government that cannot balance watermark embedding capacity, perceived transparency and anti-attack robustness. By embedding encrypted logo singular values, arithmetic coded text signatures and run-length coding compressed identity information in the three-layer decomposition of DWT, and using Convolutional Neural Networks (CNN) to achieve reversible recovery of the carrier image, the secure embedding of large-capacity watermarks is achieved, and the quality of carrier recovery and the reliability of watermark extraction are optimized [10]. V P Venkatesh et al. proposed a separable and reversible data hiding method based on the space for maneuvering after encryption and the difference in encrypted pixels to address the problems that embedded information in steganography is prone to damage to digital media and that traditional methods are difficult to balance lossless recovery and independent extraction. By combining homomorphic encryption and encrypted pixel difference techniques, a high embedding rate of 1.2 bpp was achieved, and lossless and separable processing of secret data extraction and original image restoration were realized independently [11]. The summary table of literature review is shown in Table 1.

**Table 1:** Summary table of literature review.

Methods Used	Approved metrics	Objective	Limitations	Application	References
DWT + AES encryption	PSNR = 47.50 dB; NC = 0.9882–0.9990 under Gaussian filtering, average filtering, median filtering, salt-and-pepper noise, Gaussian noise, cropping, rotation, histogram processing, and compression attacks	Embed encrypted patient data into encrypted medical images while improving imperceptibility and robustness	Mainly designed for medical image watermarking; embedding capacity and cross-domain applicability remain limited	Medical image watermarking, telemedicine	R. Singh et al. [4]
DL-based steganography in HDR image mantissa	Capacity, security, and robustness were reported to outperform existing hiding-image-in-image methods under HDR/OpenEXR settings	First to apply DL to HDR steganography with embedded LDR secret images	High network complexity; restricted to HDR (OpenEXR) format	HDR image security and visual protection	Y. Huo et al. [5]
Minimum bit variance + hashing-based double encryption	Security-oriented evaluation based on encrypted message embedding and secondary hash-based protection	Improve secure data transmission by encrypting secret messages before embedding them into cover images	Mainly review-oriented and rule-based; lacks end-to-end extraction design and directly comparable image-quality or robustness indicators	Secure communication and image-based data encryption	A. Alenizi et al. [6]
LSB embedding + GA optimization	SSIM = 0.9960–1.000 for stego and extracted images	Embed multiple color images into a single monochrome carrier image while improving embedding capacity and transmission security	Weak robustness against strong compression, noise, or geometric distortion; mainly depends on LSB-domain embedding rules	High-capacity steganographic image transmission	P. Naveen et al. [7]
Separable encrypted image reversible data hiding with histogram-free shifting and additive homomorphic encryption	Embedding capacity = 0.691 bpp	Achieve separable data extraction and lossless carrier recovery in encrypted images while improving information security	Limited compression/noise robustness	Secure image communication	R Anushiadevi et al. [8]
Hybrid image hiding based on integer wavelet transform and singular value decomposition	MSE = 9.6698; BER = 0.7205; PSNR = 29.5665 dB; SSIM ≈ 1; NCC ≈ 1	Improve the imperceptibility and robustness of hidden images within a cover image	High transform-domain computational cost; image quality and robustness may still be affected under severe distortions	Secure image transmission, defense communications, medical image confidentiality, forensic authentication, and digital rights protection	M Chinnusami et al. [9]
Three-layer DWT-based feature-dependent watermarking with CNN recovery	PSNR ≈ 44 dB; SSIM > 0.99; NC ≈ 1.0; low MSE and minimal BER after attacks	Balance watermark capacity, perceptual transparency, robustness, and reversible recovery	Complex multi-module implementation	E-governance image authentication and digital identity protection	C Lakshmi et al. [10]
Separable reversible data hiding based on room reservation after encryption and encrypted pixel difference	Embedding rate = 1.2 bpp	Achieve independent secret data extraction and lossless original image recovery in encrypted images	Limited robustness against compression, noise, and geometric distortion; mainly focuses on encrypted-domain reversibility rather than complex channel robustness Encrypted image protection and secure reversible data	Encrypted image protection	V P Venkatesh et al. [11]

In summary, existing image steganography, reversible data hiding, and robust watermarking methods have made some progress in terms of embedding capacity, image imperceptibility, and carrier recovery. However, they still struggle to simultaneously achieve high capacity, low distortion, strong robustness, and low computational complexity. Traditional transform domain methods rely on manual embedding rules, cryptographic domain reversible hiding methods suffer from insufficient stability under complex interference, and some DL methods suffer from model complexity and low inference efficiency. Therefore, it is necessary to construct a lightweight end-to-end image steganography method that can improve the robustness of information recovery and computational efficiency in complex channel environments while ensuring embedding accuracy and anti-detection capabilities. In view of this, an innovative lightweight steganography framework based on the Embed to Extract (EtENet) structure, integrating improved Inception-V3 and Xception convolution modules, and combining RepVGG structure’s reparameterization mechanism, has been proposed; At the same time, a dense connection mechanism is introduced in the decoding stage to enhance feature reuse, and adversarial constraints are constructed through a discriminative network to improve the security and anti analysis ability of steganographic images. This scheme achieves improved feature extraction and embedding accuracy, accelerated network convergence speed and parameter control, as well as significantly improved robustness under JPEG compression and noise interference conditions The research aims to ensure steganography capacity and image quality while reducing model complexity, accelerating training and inference efficiency, and providing feasible solutions for the implementation of image steganography in practical secure communication and digital copyright protection applications..

## 2. Methodology

### 2.1. Image steganography design based on etenet and improved convolution block

**Fig 1:**
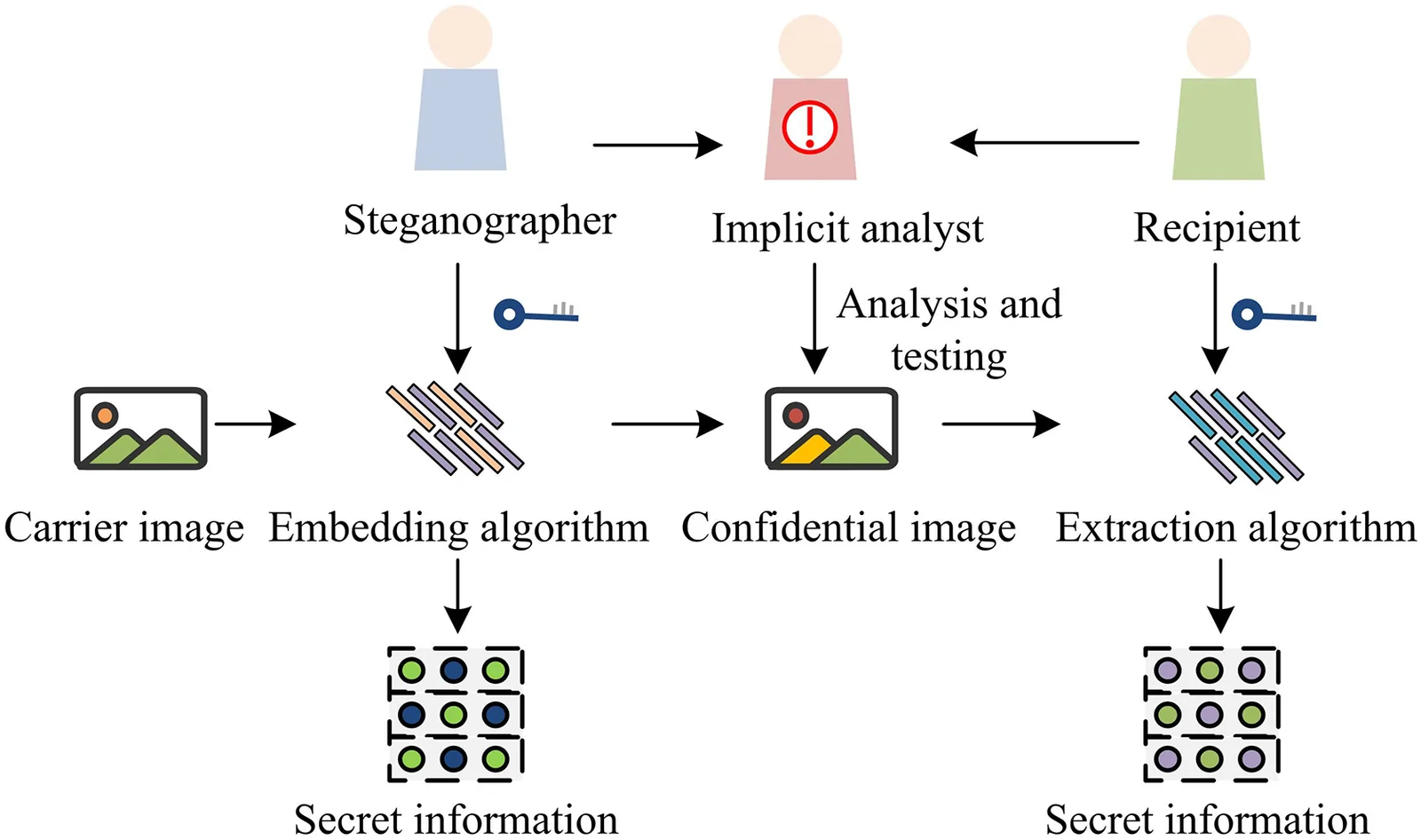
Steganography process.

With the fast advancement of the internet, big data, and artificial intelligence, social life and economic activities have become highly reliant on the collection, storage, and transmission of digital information [12]. From personal privacy protection to corporate trade secrets, data security issues are directly related to social stability and public trust [13]. Although traditional encryption techniques can effectively conceal the content of information, they struggle to mask the fact that information exists, which may trigger unnecessary suspicion or attacks in certain highly sensitive scenarios [14]. For example, in the fields of news or cross-border communication, encryption itself may lead to a crisis of trust or interception risk. In this context, steganography technology in the field of social science and engineering technology has become inevitable. Steganography can not only simply hide information, but also be applied to many social problems, such as laws and regulations and public order and good customs. Steganography technology can realize the covert transmission of sensitive information without changing social cognitive needs and affecting public interests. It has been widely concerned and gradually applied in financial regulation, intellectual property protection, digital media copyright management and other fields [15]. Therefore, steganography can not only help the development of information science, but also play an innovative auxiliary role in social science. Its core idea is to embed secret information into digital carriers in such a way that external observers find it challenging to detect anomalies in the carriers, while ensuring that the receiving end can accurately extract the hidden information. A steganographic system typically consists of three participants: the steganographer, the steganalyst, and the receiver. These parties interact through the processes of “embedding-detection-extraction,” as illustrated in Fig 1 [16].

**Fig 2:**
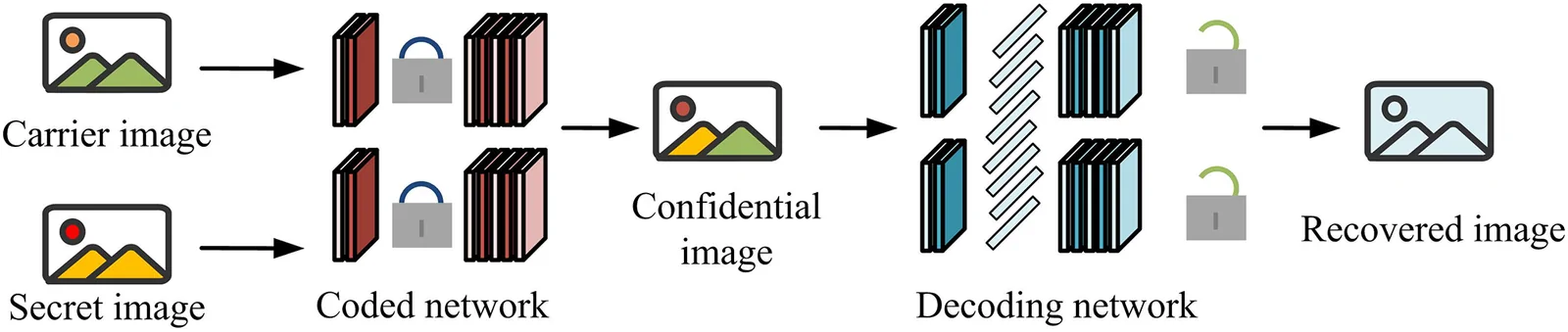
EtENet model structure.

In Fig 1, the steganographer inputs the carrier image and the secret information to be concealed into the embedding algorithm and generates a stego image by incorporating a cryptographic key. The generated stego image can, on one hand, be transmitted to the receiver and, on the other hand, may be subjected to detection by the steganalyst to determine whether it contains secret information. After obtaining the stego image, the receiver successfully recovers the hidden secret information through the extraction algorithm combined with the cryptographic key [17]. Building upon this, the study further proposes an implementation approach based on an end-to-end architecture, namely the EtENet model, with the overall framework illustrated in Fig 2.

In Fig 2, the model takes the carrier image and the secret image as inputs. Through the encoding network, the secret information is embedded into the carrier image to generate a stego image. Subsequently, the stego image is fed into the decoding network to extract and recover the embedded secret image, thereby accomplishing the complete process of image steganography and information extraction. To balance model performance and efficiency during this process, convolutional modules are introduced into the network architecture. Inception-V3 adopts a parallel multi-scale convolution structure for extracting image features at different spatial scales, which is beneficial for embedding steganographic information in different texture levels without causing significant edge distortion and improving the imperceptibility of embedding. Inception-V3 uses 1 × 1 convolutions for dimensionality reduction, as demonstrated in equation (1) [18].


$$\begin{array}{c} F_{p,q}=\sum_{c=0}^{C_{out}-1}Z_{p,q,c}\theta_{c} \end{array}$$ (1)

In equation (1), F is the output characteristic graph, Z is the input characteristic graph, (p,q) is the spatial position index, $\theta_{c}$ is the convolution kernel parameter, and $C_{out}$ is the number of output channels. Dimension reduction is achieved by reducing the $C_{out}$ of the output characteristic graph, which effectively reduces the amount of calculation and model complexity, as shown in equation (2) [19].


$$\begin{array}{c} F_{p,q}=\sum_{u=0}^{U-1}\sum_{v=0}^{V-1}\sum_{c=0}^{C_{in}-1}Z_{p+u,q+v,c}\theta_{u,v,c} \end{array}$$ (2)

In equation (2), U×V is the size of convolution kernel, (u,v) is the spatial index of convolution kernel, and $C_{in}$ is the number of input channels. Xception introduces deep separable convolution on the basis of perception-V3, and decomposes the standard convolution into deep convolution and pointwise convolution, significantly reducing the amount of model parameters. Xception adaptively processes the features of different channels, which helps to control the embedding strength of different channels, improve the ability to regulate feature distribution, and enhance embedding flexibility while reducing computational complexity. Structural re parameterization realizes parameter compression through linear transformation of the network, which helps to boost the efficiency of training and reasoning, and enhance the generalization performance of the model. Input the characteristic diagram into the neural network composed of convolution layer and Batch Normalization (BN) layer, and its calculation process is shown in equation (3) [20].


$$\begin{array}{c} F^{\prime }=\frac{\alpha \theta }{\sqrt{s^{\text{2}}+R}}Z+\beta +\frac{\alpha }{\sqrt{s^{\text{2}}+R}}(b-\mu ) \end{array}$$ (3)

In equation (3), $F^{\prime }$ is the output characteristics after fusing convolution and BN, α is the scaling coefficient of BN layer, s represents the variance of BN layer, and R is a constant to prevent the denominator from being zero. β is the translation coefficient of BN layer, μ is the average value of BN layer, and b is the convolution offset. Through structural re-parameterization, the updated weight and bias parameters can be derived, and the expression is shown in equation (4) [21].


$$\begin{array}{c} \left\{ \begin{array}{c} @l@\theta^{\prime }=\frac{\alpha \theta }{\sqrt{s^{\text{2}}+R}}\\ b^{\prime }=\beta +\frac{\alpha \theta }{\sqrt{s^{\text{2}}+R}}(b-\mu ) \end{array}\right. \end{array}$$ (4)

**Fig 3:**
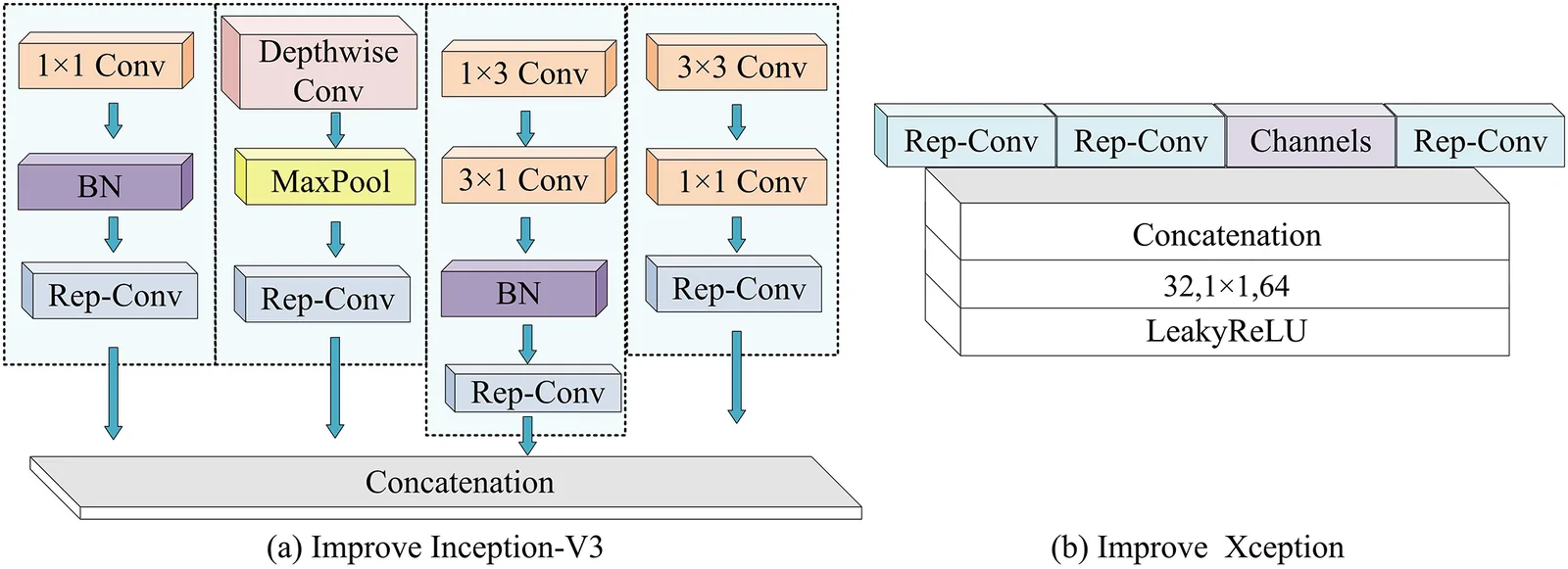
Improved convolutional block structure.

In equation (4), $\theta^{\prime }$ and $b^{\prime }$ are the parameterized equivalent weights and bias terms respectively. To reduce the complexity of image steganography models and enhance feature extraction precision, the study introduces RepVGG modules based on the architectures of Inception-V3 and Xception. RepVGG allows the use of multi branch structures to fully extract high-order spatial channel combination features during the training phase, while in the inference phase, it is transformed into an equivalent single 3 × 3 convolution structure through structural reparameterization, significantly reducing the number of parameters and computational overhead [22]. Unlike the depthwise separable convolution model, the parameter conversion process of RepVGG is a strict equivalent linear transformation, which does not lose feature expression ability and is therefore more conducive to robust feature recovery during steganography. By integrating the multi-scale feature extraction of Inception-V3 and the depthwise separable convolutions of Xception, the improved convolutional block enhances joint modeling of spatial and channel features. Furthermore, the incorporation of BN and residual connections accelerates network convergence and improves stability. The structure of the improved convolutional block is illustrated in Fig 3.

Fig 3(a) and 3(b) depict the improved convolutional blocks for Inception-V3 and Xception, respectively. In Fig 3(a), after incorporating RepVGG convolutional units, Inception-V3 includes a BN layer in each channel to achieve BN, thereby accelerating convergence in deep networks. Meanwhile, all branches employ RepVGG structures for convolutional operations, with the last branch utilizing two RepVGG blocks to replace the traditional 5 × 5 convolutional kernel. This approach effectively reduces the model parameter count while preserving the receptive field [23]. In Fig 3(b), Xception applies RepVGG modules to each channel, achieving channel dimensionality reduction through 1 × 1 convolutions and introducing a residual connection mechanism that adds the input directly to the output. This enhances feature representation while improving training stability. By combining the improved Inception-V3 and Xception convolutional blocks, the system can more comprehensively capture spatial and channel-wise information in images, significantly enhancing feature extraction capabilities and image reconstruction quality. The study found that the structural reparameterization strategy of RepVGG can effectively reduce computational overhead during the inference phase while still maintaining the ability to model high-order spatial channel features. During the training phase, the network retains a multi branch convolutional structure to achieve parallel extraction of multi-scale features, thereby fully capturing the contextual associations between the carrier image and secret information; In the inference stage, each branch is strictly equivalently fused into a single 3x3 convolution structure through linear transformation, significantly reducing the number of parameters and floating-point operations. Due to the fact that the fusion process belongs to a mathematical equivalent mapping, feature information will not be lost. Therefore, even under complex compression or noise interference conditions, the model can still maintain stable recovery ability for hidden data.

### 2.2. Coding network based on lightweight convolutional block

**Fig 4:**
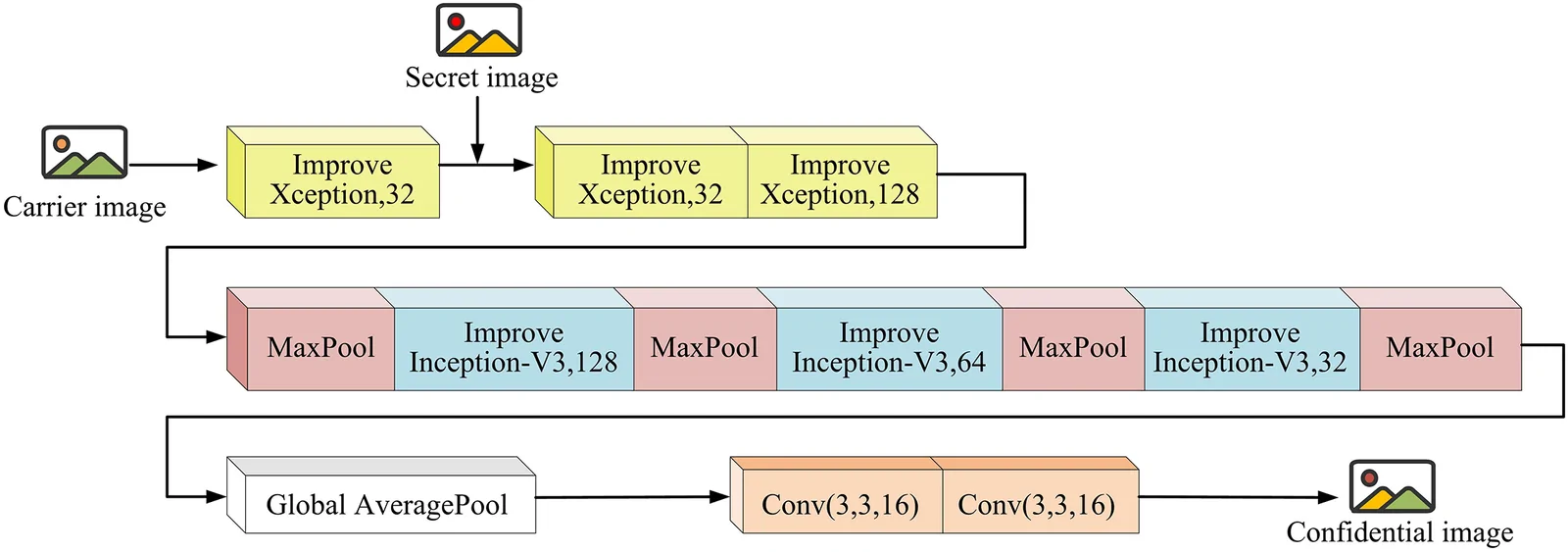
Encoding network structure.

Building on the EtENet framework and the improved convolutional blocks, the study further integrates these modules into the encoding network—a critical component of the steganographic system. The encoding network serves as the core within the end-to-end steganography framework, with its primary task being the effective embedding of secret images into carrier images while minimizing disruptions to the carrier’s appearance, thereby ensuring the imperceptibility of stego images. At the design level of image steganography, this process requires balancing embedding capacity and image quality while ensuring that the generated stego images exhibit robustness against compression and interference [24]. Unlike traditional rule-based or handcrafted feature embedding methods, the encoding network designed in this study fully leverages the feature extraction capabilities and parameter efficiency of lightweight convolutional blocks. This enables deep modeling of multi-level spatial and channel features while maintaining controllable model complexity. To further reduce losses of secret information during transmission, the study introduces residual connections into the encoding architecture, allowing shallow-layer features to be directly transferred to deeper networks. This effectively preserves original feature information and enhances feature propagation. The structure of the encoding network is presented in Fig 4.

In Fig 4, the network consists of three improved Inception-V3 convolutional blocks, three improved Xception convolutional blocks, and three basic convolutional blocks. After the secret image undergoes processing through multiple layers of improved Xception blocks, its features are continuously transmitted to subsequent network layers via residual connections, effectively minimizing losses of secret information during transmission. Meanwhile, the carrier image, after global average pooling and convolutional processing, participates in subsequent fusion and encoding alongside the features of the secret image [25]. Given that the improved Inception-V3 blocks incorporate deeper hierarchical structures with gradually increasing channel counts during stacking, MaxPool operations are introduced after each improved Inception-V3 block to reduce model width and channel numbers, thereby alleviating computational complexity and accelerating network convergence. Ultimately, the carrier image, after convolutional and fusion processing, is transformed into a stego image, achieving effective embedding of secret information. To enhance the security of information hiding, the encoding network leverages the training process to learn embedding information across diverse spatial structures or texture features, with calculations as shown in equation (5) [26].


$$\begin{array}{c} F_{h}=E\left(concat\left(F_{c},F_{s}\right)\right) \end{array}$$ (5)

**Fig 5:**
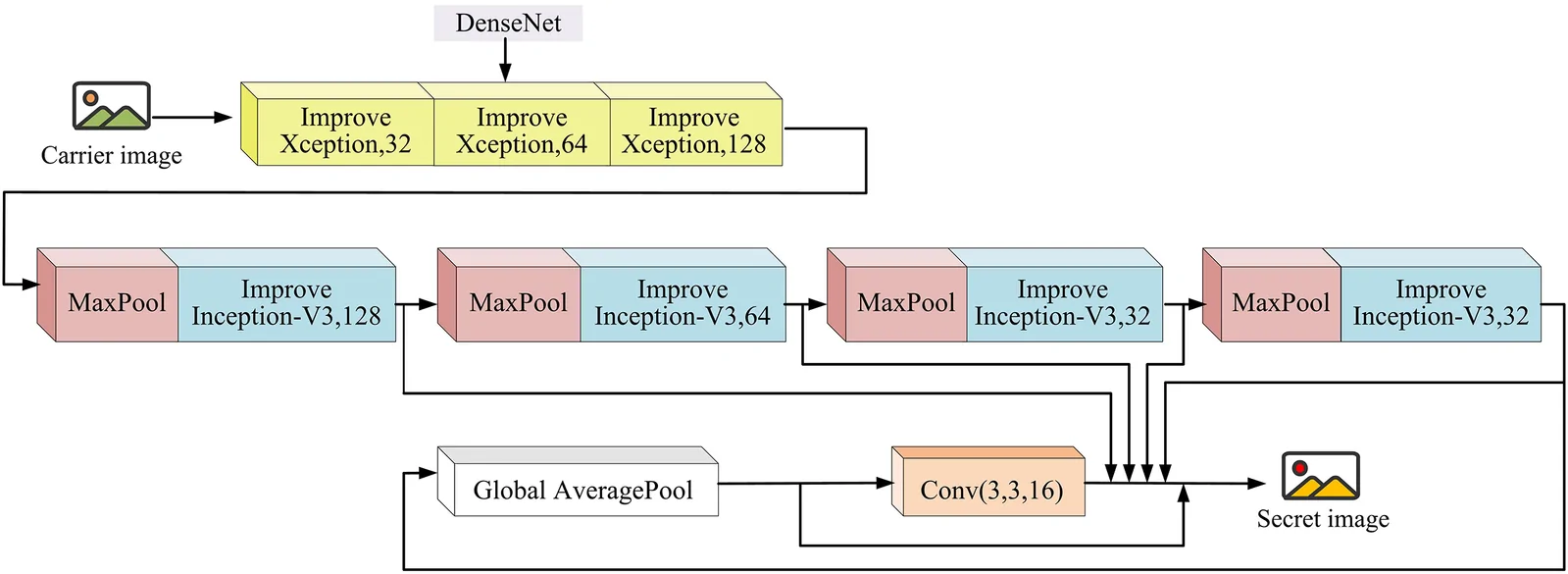
Decoding network structure.

In equation (5), $F_{h}$ represents the secret image, $F_{c}$ represents the carrier image, $F_{s}$ represents the secret image, and E represents the encoding network. The adaptive capability of the network model exhibits certain limitations, resulting in suboptimal performance in image feature extraction. The DenseNet dense connection mechanism alleviates gradient decay and promotes the propagation of shallow embedded features to deep layers through cross layer feature transfer, which is beneficial for improving the accuracy of secret information recovery. Compared to a single residual structure, Dense connections can avoid key information being weakened in deep convolution. To address this, the study employs a DenseNet structure to enhance feature reuse and mitigate gradient vanishing. The structure of the decoding network is presented in Fig 5.

In Fig 5, the decoding network takes the stego image as input. Initially, it extracts deep features through multiple layers of improved Xception convolutional blocks and pooling operations, while gradually compressing the channel dimensions. The introduction of dense connections within the network enables direct transmission of features extracted from shallow layers to deeper layers, thereby facilitating multi-level feature reuse and effectively enhancing the recovery quality of the secret image. At the same time, the synergistic effect of dense connections and residual paths helps to improve feature preservation during embedding and extraction processes. Dense connections enable shallow information to be directly transmitted to deep networks through cross layer feature reuse mechanisms, avoiding gradient vanishing and reducing feature decay issues; The residual path provides an identity mapping channel to avoid feature blurring caused by deep convolution stacking, allowing the network to maintain its original structural information during deep modeling. To prevent the model from overly relying on shortcut features and causing overfitting, dropout and weight regularization strategies are introduced during the training process to constrain the model capacity and enhance the stability of feature learning. Therefore, this structural design can effectively improve the embedding and Recovery Accuracy (RA) of steganographic information without increasing the risk of overfitting. RA refers to the degree of restoration at the pixel level between the decoded secret image and the original secret image. As the layer count increases, the model parameter count may rise significantly; hence, an improved lightweight convolutional module is incorporated into the decoding structure to effectively control model complexity and accelerate training convergence. Finally, after global average pooling and convolutional reconstruction layers, the network outputs the recovered secret image. The recovery of the secret image through the decoding network is calculated as shown in equation (6) [27].


$$\begin{array}{c} F_{rec}=G\left(F_{h}\right) \end{array}$$ (6)

In equation (6), $F_{rec}$ is the recovered secret image, and G is the decoding network. After the embedding and extraction of secret information are completed, it remains necessary to further verify the concealment and security of the stego image. To this end, the study introduces a discriminative network within the EtENet framework, which is employed to classify and judge input images, distinguishing whether they are carrier images or stego images. By incorporating a discriminator, adversarial constraints can be imposed on the encoding network during the training process, compelling it to generate stego images that are visually closer to the original carrier images, thereby effectively enhancing the robustness and anti-detection capabilities of the steganographic algorithm [28]. To avoid model collapse caused by strong discriminators or unstable updates during adversarial training, multiple balancing strategies were adopted in the design of adversarial structures. Firstly, in the early stages of training, a lower learning rate is used for the discriminator, and the adversarial loss weights are gradually increased, so that the encoding network can learn the basic embedded features first in the early stages and avoid being impacted by too strong adversarial constraints. Secondly, an alternating update mechanism of discriminator and encoder decoder network is adopted to reduce the risk of gradient fluctuations. At the same time, the joint introduction of image fidelity loss and perceptual loss in the training target suppresses excessive embedding behavior, ensures the refinement of embedded features, and does not damage the overall image feature distribution. The total loss function is shown in equation (7).


$$\begin{array}{c} \left\{ \begin{array}{c} @l@L_{total}=\lambda_{\text{1}}L_{rec}+\lambda_{\text{2}}L_{cover}+\lambda_{\text{3}}\left(t\right)L_{adv}\\ \lambda_{\text{3}}\left(t\right)=min\left(\lambda_{\text{3}}^{max},\frac{t}{T_{warm}},\lambda_{\text{3}}^{max}\right) \end{array}\right. \end{array}$$ (7)

**Fig 6:**
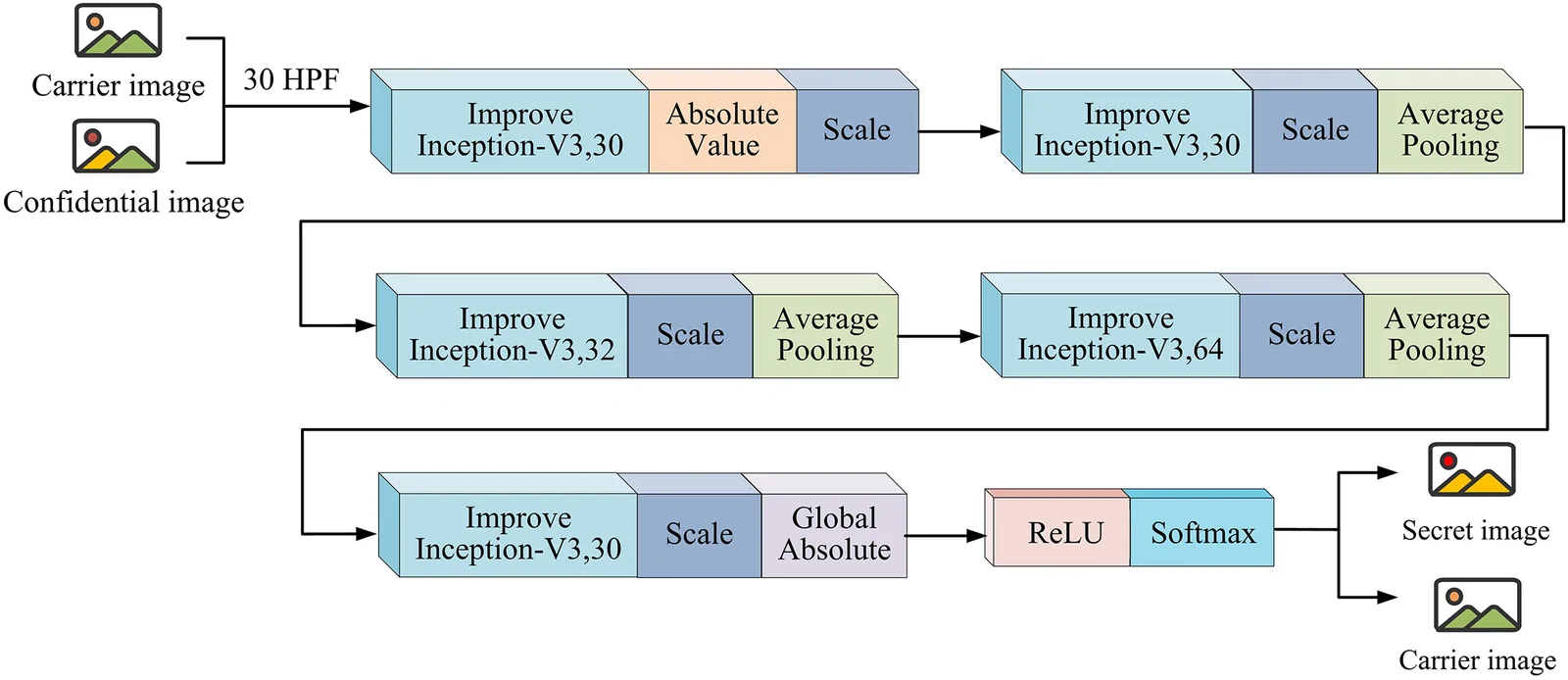
Distinguish network structure.

In equation (7), $L_{total}$ is the total loss function, $L_{rec}$ represents the secret image reconstruction loss, $L_{cover}$ represents the steganographic image fidelity loss, and $L_{adv}$ represents the adversarial loss fed back by the discriminator. $\lambda_{\text{1}}$ and $\lambda_{\text{2}}$ are fixed weights for reconstruction loss and fidelity loss, respectively, while $\lambda_{\text{3}}\left(t\right)$ represents the adversarial loss weight that gradually increases with training iterations. t is the current training round, and $T_{warm}$ represents the threshold for enabling adversarial training. The structure of the discriminative network is presented in Fig 6.

In Fig 6, the discriminative network takes both stego images and carrier images as inputs. Initially, it applies high-pass filtering to the stego images, followed by operations such as convolutional blocks, absolute value computation, scaling transformation, and average pooling to progressively extract discriminative features. Meanwhile, the carrier images also undergo convolutional blocks and pooling layers to obtain corresponding feature representations. After further convolution, scaling adjustment, and global absolute value calculation, the features from both pathways are fed into an activation function and a Softmax classifier, ultimately producing an output for judgment [29]. The study introduces a discriminative network into the EtENet framework and optimizes the convolutional blocks. Additionally, it proposes an Image Steganography Algorithm Based on EtENet and Lightweight Convolutional Network (ISA-ELCN), the pseudocode is shown in Algorithm 1:

Algorithm 1: Training procedure of ISA-ELCN

Input:

 Training set of carrier images {C_i} and secret images {S_i}

 Max training epochs T, batch size B, learning rate η

 Initialized parameters θ_enc, θ_dec, θ_dis for encoder, decoder and discriminator

Output:

   Trained encoder E_enc(·; θ_enc) and decoder E_dec(·; θ_dec)

1: for epoch = 1 to T do

2:   Shuffle the training set

3:   for each mini-batch {(C_i, S_i)}_{i = 1}^B do

4:     # ---------- Forward embedding and extraction ----------

5:     Stego images: ŜC_i = E_enc(C_i, S_i; θ_enc)

6:     Recovered secrets: ŜS_i = E_dec(ŜC_i; θ_dec)

7:     # ---------- Compute reconstruction and perceptual losses ----------

8:     L_rec = ReconstructionLoss(S_i, ŜS_i) # e.g., MSE, MAE

9:     L_cover = CoverFidelityLoss(C_i, ŜC_i) # e.g., PSNR/SSIM-based

10:     # ---------- Discriminator forward and adversarial loss ----------

11:     y_real = D(C_i; θ_dis) # should be classified as cover

12:     y_fake = D(ŜC_i; θ_dis) # should be classified as stego

13:     L_dis  = DiscriminatorLoss(y_real, y_fake) # e.g., BCE loss

14:     L_adv  = AdversarialLoss(y_fake) # used to guide encoder

15:     # ---------- Update discriminator ----------

16:     θ_dis ← θ_dis − η· ∇_{θ_dis}(L_dis)

17:     # ---------- Update encoder and decoder ----------

18:     L_total = λ1·L_rec + λ2·L_cover + λ3·L_adv

19:     θ_enc  ← θ_enc − η· ∇_{θ_enc}(L_total)

20:     θ_dec  ← θ_dec − η· ∇_{θ_dec}(L_total)

21:    end for

22: end for

23: return θ_enc, θ_dec

**Fig 7:**
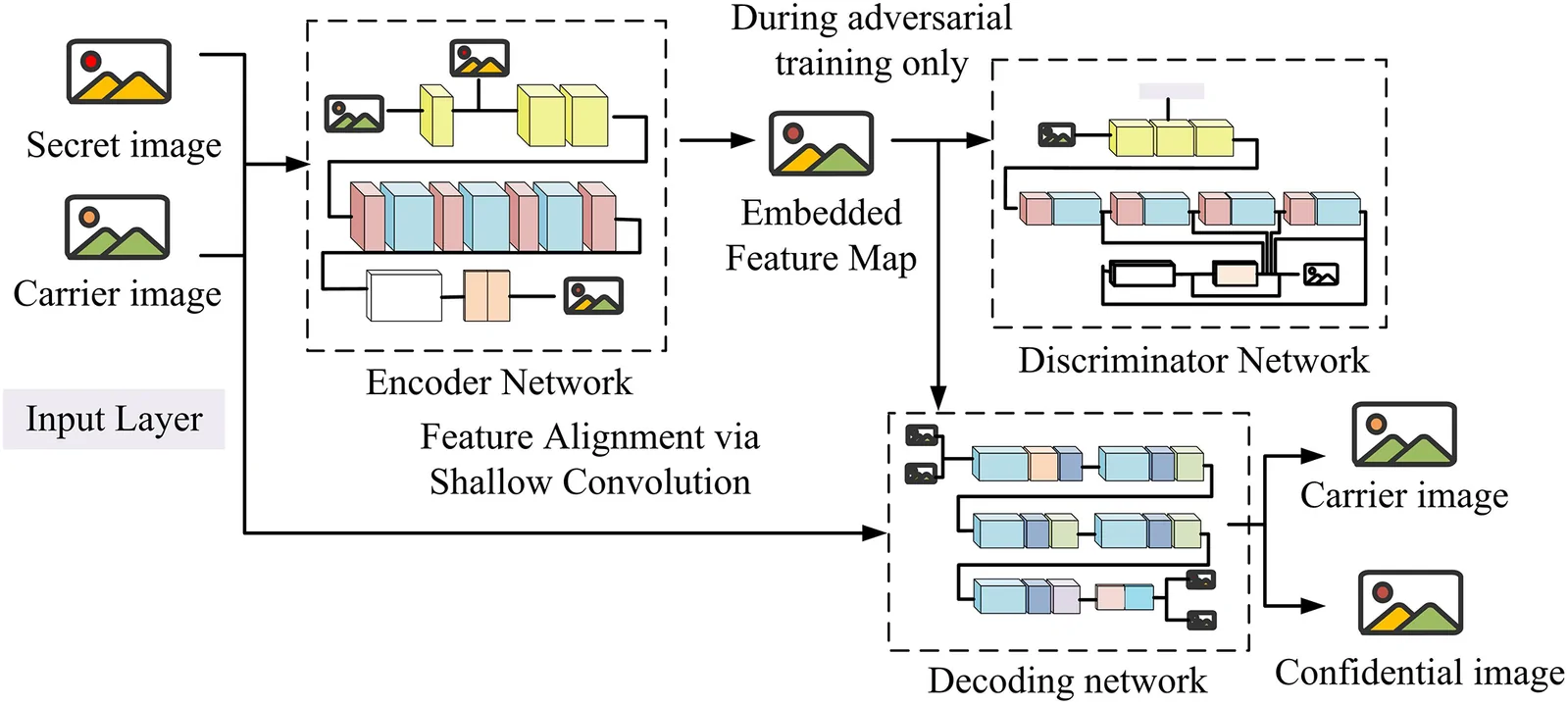
ISA-ELCN process.

The ISA-ELCN structure is shown in Fig 7.

In Fig 7, the algorithm primarily consists of three components: an encoding network, a decoding network, and a discriminative network. The encoding network combines secret images with carrier images to generate stego images, while the decoding network extracts and reconstructs secret information from the stego images. Meanwhile, the discriminative network is introduced within the EtENet framework to distinguish between carrier images and stego images, thereby enhancing the model’s robustness and concealment. The algorithm introduces a lightweight module and optimizes the convolution structure. The model is clearer and simpler, the convergence speed is faster, and the embedding accuracy and security of secret information are guaranteed. The algorithm embedding and extraction of pseudocode are shown in Algorithm 2.

Algorithm 2: Embedding and extraction procedure

Input:

  Trained encoder E_enc(·; θ_enc) and decoder E_dec(·; θ_dec)

  Carrier image C, secret image S

Output:

  Stego image C_stego, recovered secret image S_rec

# ---------- Embedding stage ----------

1: Preprocess carrier image C and secret image S

2: C_stego = E_enc(C, S; θ_enc)

3: Transmit or store C_stego through a public channel

# ---------- Extraction stage ----------

4: Receive (possibly distorted) stego image C_stego’

5: Preprocess C_stego’ to match the training resolution and normalization

6: S_rec = E_dec(C_stego’; θ_dec)

7: Post-process S_rec if necessary (e.g., de-normalization, rounding)

8: return C_stego, S_rec

## 3. Results and analysis

### 3.1. Performance test of ISA-ELCN image steganography algorithm

To evaluate the validity and dependability of ISA-ELCN algorithm in operation, and ensure that the experimental results have practical significance, this study constructed an experimental environment based on DL. The experiments were conducted on a host equipped with an Intel Core i7-12700K processor, an NVIDIA RTX 3080 GPU, and 32 GB of memory, running on Ubuntu 22.04 LTS. Python 3.9 was used as the primary programming language, and PyTorch 1.13 served as the DL framework. Select the BOSSBase 1.01 dataset for the experiment, which contains 10000 uncompressed natural grayscale images with a resolution of 512 × 512 pixels. The images cover various real scenes such as natural landscapes, buildings, and portraits, with rich texture and structural features, which can be used to comprehensively evaluate the performance of steganography algorithms in information hiding and extraction. Before the experiment, all images were converted to grayscale images, normalized to the [0,1] interval, and uniformly adjusted to 256 × 256 pixels to reduce computational overhead. To enhance the generalization ability of the model, data augmentation strategies such as horizontal and vertical flipping, Gaussian noise injection, and JPEG compression simulation were also used during the training process. Ablation experiments were conducted on the BOSSBase 1.01 dataset, using bit error rate (BER) and the area under the curve (Steganalysis AUC) as evaluation metrics. Steganalysis AUC characterizes the detector’s ability to distinguish between the carrier image and the steganalyte image, rather than the classification performance of the proposed model; a lower AUC value indicates that the steganalyte image is more difficult to detect, and the method has stronger stealth and anti-detection capabilities. The ablation experiment results are shown in Table 2.

**Table 2:** Outcomes of ablation experiment.

Method	BER	Steganalysis AUC
w/o Improved Inception-V3 Convolutional Block	0.018	0.522
w/o Improved Xception Convolutional Block	0.020	0.554
w/o Densely connected	0.022	0.573
w/o Discriminant network	0.011	0.611
ISA-ELCN	0.008	0.398

**Fig 8:**
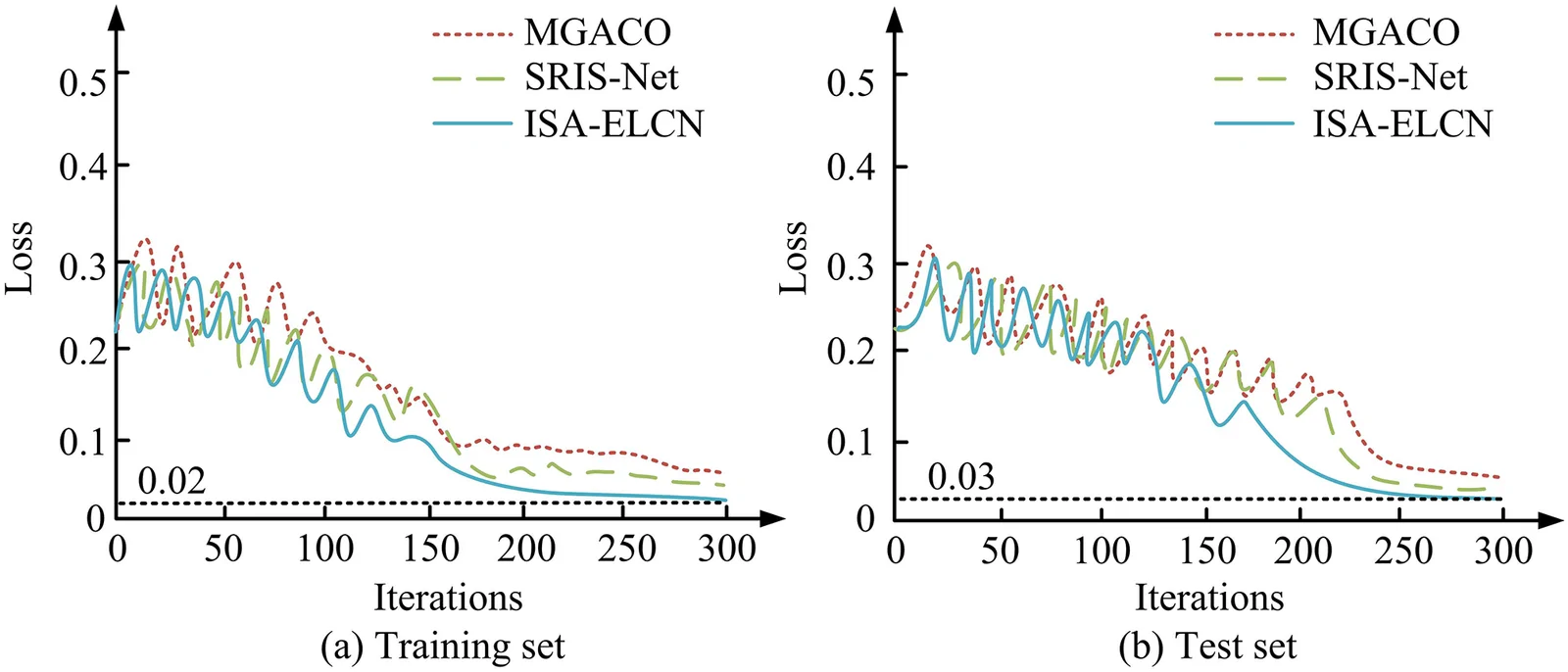
Loss function variation curve.

According to Table 2, the complete ISA-ELCN algorithm demonstrated optimal performance in both BER and Steganalysis AUC metrics, achieving a balance between the RA of secret information and concealment. When the improved Inception-V3 convolutional blocks were removed, the BER increased to 0.018, and the Steganalysis AUC rose to 0.522, indicating that the multi-branch structure and dimensionality reduction mechanism played a crucial role in reducing information loss and enhancing feature representation. Similarly, after removing the improved Xception convolutional blocks, the BER and Steganalysis AUC increased to 0.020 and 0.554, respectively, further highlighting the importance of depthwise separable convolutions for effective feature extraction. Additionally, when dense connections were not employed at the decoding end, the transmission and reuse capabilities of secret information were weakened, resulting in a BER of 0.022 and a Steganalysis AUC of 0.573. This reflected the significant role of cross-layer feature fusion in enhancing RA. In contrast, although the BER remained relatively low after removing the discriminative network, the Steganalysis AUC increased to 0.611, demonstrating the critical importance of the discriminator in improving steganographic resistance to detection. Overall, the various modules of ISA-ELCN worked in synergy, not only ensuring high-precision recovery of secret images but also effectively enhancing the security and robustness of steganography. To further evaluate the algorithm’s convergence performance and training stability, the dataset was first divided into training and testing sets in an 8:2 ratio. The proposed algorithm was then compared with a steganalysis algorithm based on Modified Graph Clustering Based Ant Colony Optimization (MGACO) feature selection and a random forest classifier [30], as well as a robust image steganography algorithm called The Secure and Robust Image Steganography Network (SRIS-Net) [31]. The loss function variation curves for these comparisons are shown in Fig 8.

**Fig 9:**
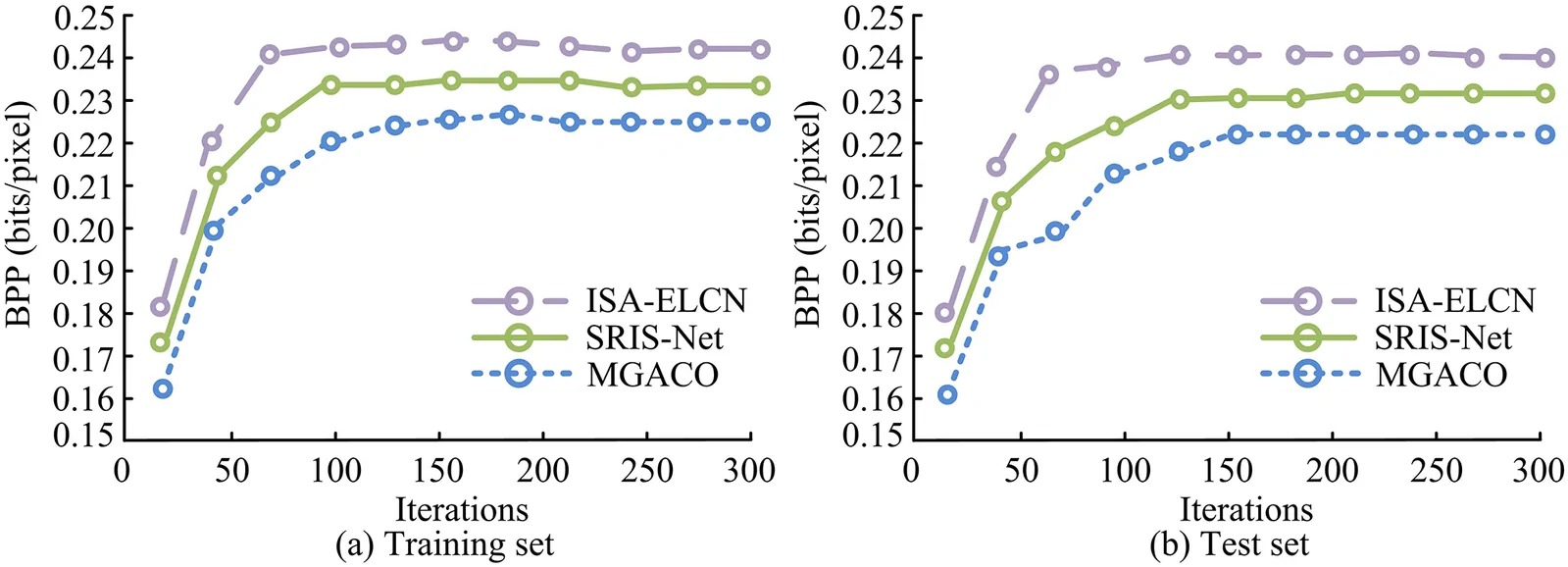
BPP variation results of different algorithms.

Fig 8(a) and 8(b) respectively illustrate the trends of loss curves for the three methods on the training and testing sets as the number of iterations increased. In Fig 8(a), as the number of iterations grew, the losses gradually converged, with ISA-ELCN exhibiting the fastest decline rate. After convergence, its loss value approached 0.02, significantly lower than SRIS Net’s 0.04 and MGACO’s 0.09, indicating superior convergence and fitting capabilities during the training phase. In Fig 8(b), all methods similarly demonstrated a trend of gradually decreasing loss values, but the differences were more pronounced. ISA-ELCN achieved a notably lower loss value than the other comparative methods after 200 iterations, ultimately stabilizing at around 0.03. In contrast, SRIS-Net and MGACO exhibited slower convergence and greater fluctuations in their loss values. This indicated that ISA-ELCN not only demonstrated excellent optimization efficiency during training but also exhibited stronger generalization ability during testing, effectively reducing the risk of overfitting and achieving superior overall performance. The variations in Bits Per Pixel (BPP) for various algorithms on the dataset are presented in Fig 9.

Fig 9(a) and 9(b) respectively depict the changes in BPP for the three methods on the training and testing sets. In Fig 9(a), the BPP values for all three methods were relatively low during the initial iterations. As the iteration count increased, the BPP values for all methods significantly rose and stabilized after 100 iterations. Specifically, ISA-ELCN ultimately stabilized at 0.245, SRIS-Net at approximately 0.234, and MGACO at around 0.226. The trend in Fig 9(b) was generally consistent with that of the training set, albeit at slightly lower overall levels. All three methods showed rapid increases in the first 50 iterations. ISA-ELCN converged to 0.241 after 100 iterations, while SRIS-Net converged to around 0.230, and MGACO ultimately stabilized near 0.223. Overall, ISA-ELCN demonstrated faster convergence speeds and higher BPP values on both the training and testing sets, indicating its superiority over SRIS-Net and MGACO in regard to information embedding capacity and generalization ability. To comprehensively evaluate the quality of stego images, the study selected Peak Signal-to-Noise Ratio (PSNR), SSIM, and Learned Perceptual Image Patch Similarity (LPIPS) as evaluation metrics, with the results presented in Table 3.

**Table 3:** Image quality test results.

Data set	Method	PSNR (dB)	SSIM	LPIPS
Training set	MGACO	37.8	0.945	0.082
	SRIS-Net	39.5	0.958	0.067
	ISA-ELCN	41.2	0.971	0.052
Test set	MGACO	37.2	0.940	0.086
	SRIS-Net	39.0	0.954	0.071
	ISA-ELCN	40.7	0.969	0.055

As observed from the results in Table 3, on both the training and testing sets, the image quality of ISA-ELCN outperformed that of SRIS-Net and MGACO. On the training set, ISA-ELCN achieved a PSNR of 41.2 dB, significantly higher than SRIS-Net’s 39.5 dB and MGACO’s 37.8 dB, indicating its stronger capability in pixel-level restoration. Meanwhile, ISA-ELCN’s SSIM reached 0.971, surpassing SRIS-Net and MGACO in structural similarity, demonstrating its ability to better preserve the structural information of the original images. In terms of the LPIPS metric, ISA-ELCN recorded the lowest value at 0.052, while SRIS-Net and MGACO scored 0.067 and 0.082, respectively, indicating that ISA-ELCN was closest to the original images in perceptual quality. The performance on the testing set was consistent with that on the training set, with ISA-ELCN achieving PSNR and SSIM values of 40.7 dB and 0.969, both significantly superior to the comparative methods. Additionally, its LPIPS value was only 0.055, still outperforming SRIS-Net and MGACO. The results demonstrated that ISA-ELCN could effectively maintain the visual quality and structural features of stego images while ensuring high-precision information embedding and extraction, exhibiting superior generalization ability and stability.

### 3.2. Application effect analysis of isa-elcn image steganography algorithm

**Fig 10:**
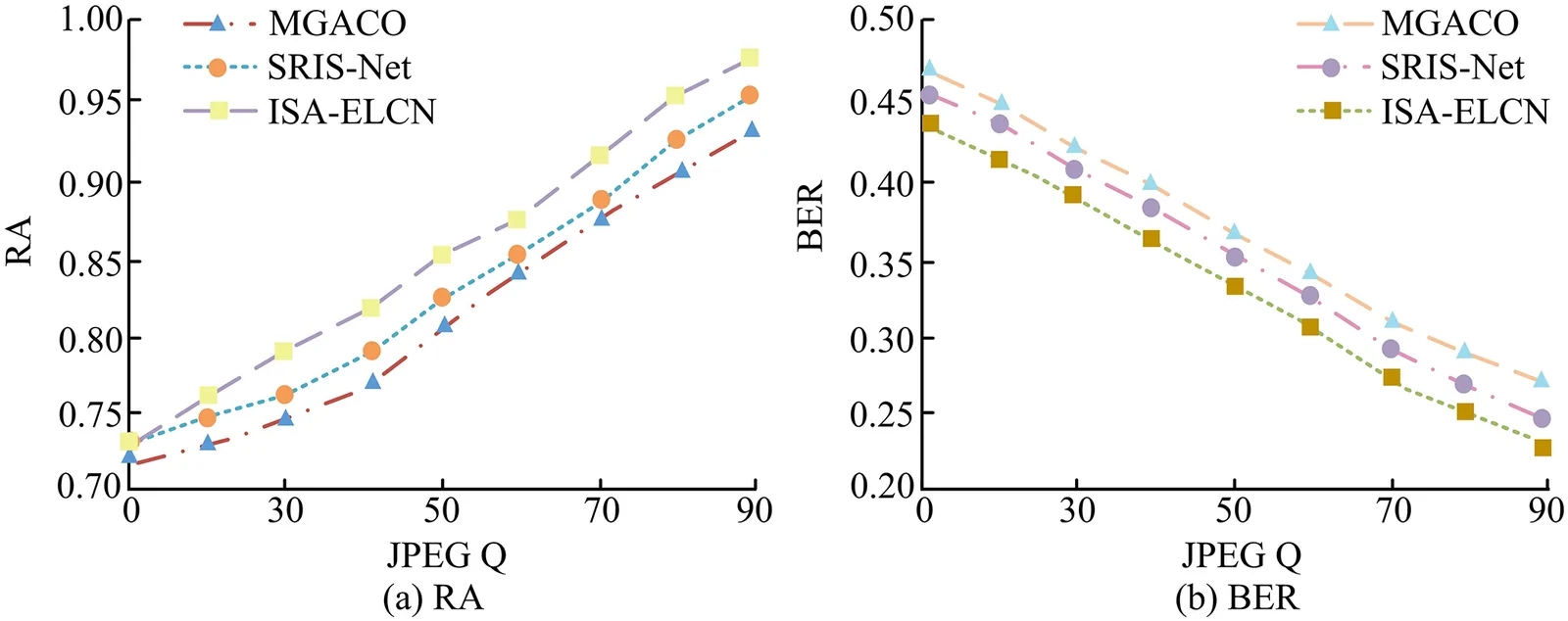
RA and BER under different JPEG compression strengths.

To verify the practicality of the proposed ISA-ELCN algorithm in complex communication environments, an analysis of its application effectiveness was conducted through simulation experiments. The experiments were carried out on the BOSSBase 1.01 dataset, with varying JPEG compression strengths set to simulate the distortions that images might encounter during transmission over public channels. The results of RA and BER under JPEG compression with different compression strengths are shown in Fig 10.

**Fig 11:**
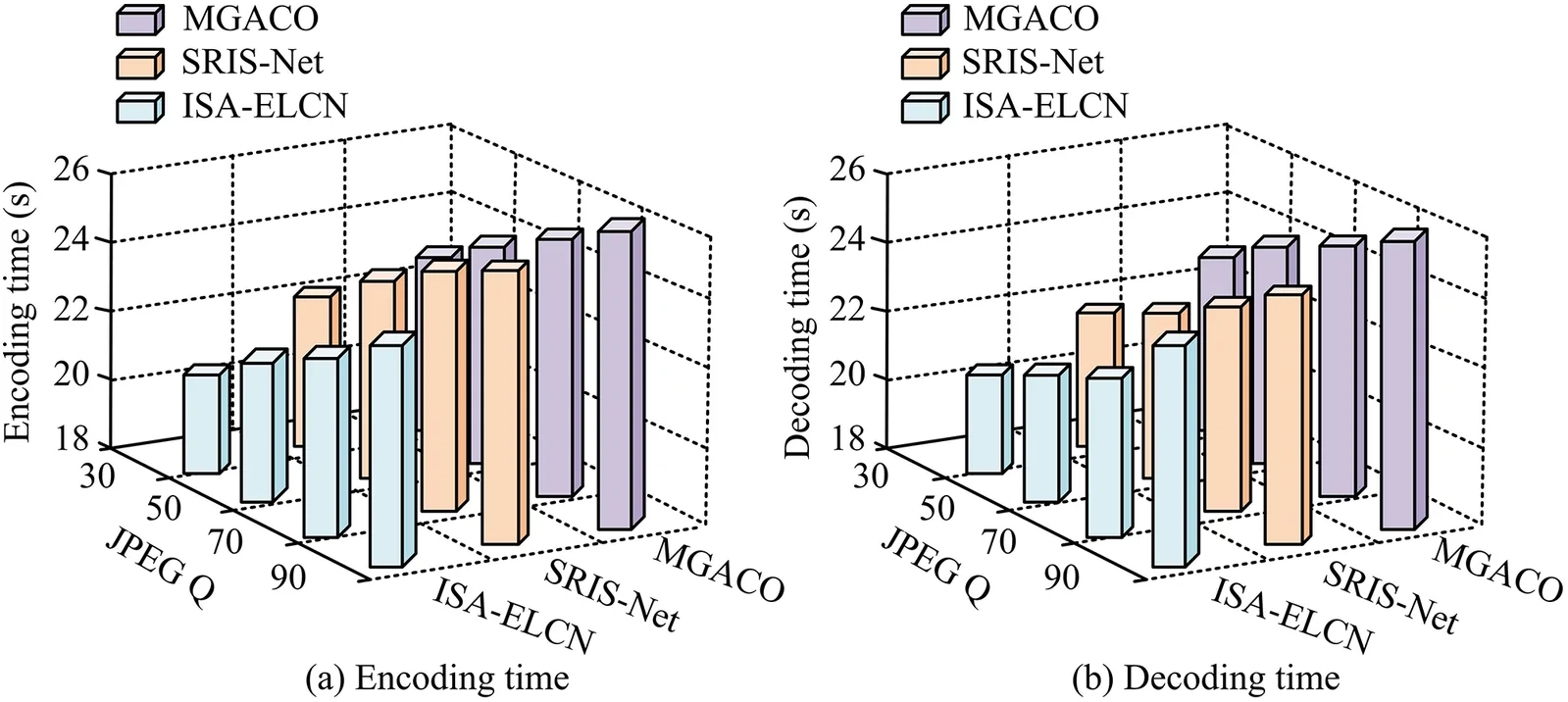
Result of changes in encoding time and decoding time.

Fig 10(a) and 10(b) respectively illustrate the trends of RA and BER for the three methods under different JPEG compression strengths as the Q value varied. As the Q value increased to 90, the RA gradually rose while the BER gradually declined, indicating that higher compression quality led to better recovery performance of steganographic information. In Fig 10(a), as the JPEG Q value increased, the RA of all three methods gradually improved, demonstrating that higher compression quality resulted in better recovery of secret information. Among them, MGACO exhibited the lowest RA across all compression strengths, while SRIS-Net showed slight improvement. In contrast, ISA-ELCN maintained the highest RA across the entire range, with a particularly pronounced advantage under strong compression conditions. At Q = 30, ISA-ELCN’s RA exceeded 0.80, whereas MGACO’s was only around 0.78. When Q = 90, ISA-ELCN’s RA approached 0.98, while SRIS-Net and MGACO reached 0.96 and 0.95. Conversely, in Fig 10(b), the BER consistently decreased as the Q value increased, indicating that improved compression quality led to a lower BER. Among the methods, MGACO consistently had the highest BER, while ISA-ELCN maintained the lowest error rate across all compression strengths. At Q = 30, ISA-ELCN’s BER was 0.38, whereas MGACO’s was close to 0.46. When Q = 90, ISA-ELCN’s BER dropped to 0.23, significantly outperforming SRIS-Net’s 0.25 and MGACO’s 0.28. ISA-ELCN can still maintain a low error rate and image structure at Q = 30, mainly due to its effective preservation of frequency domain and spatial domain correlation during the embedding stage. Firstly, the encoding network utilizes multi-scale convolution and channel modulation to map the embedded information to the relatively stable low-frequency and mid low frequency regions for JPEG quantization, avoiding excessive disturbance of high-frequency details and reducing the destruction of secret information during the quantization process; Meanwhile, EtENet enhances local correlation and intra block consistency to smoothly embed steganographic features into the texture and brightness changes of the carrier image. Even if block effects and detail loss occur after compression, the decoding network can still recover hidden information from structural correlations, thereby achieving an effective balance between BER and structural integrity. Subsequently, an analysis of the different model complexities was conducted, with the changes in encoding and decoding times shown in Fig 11.

Fig 11(a) and 11(b) respectively depict the changes in encoding and decoding times for the three methods under different JPEG compression quality factors. As shown in Fig 11(a), in terms of encoding time, MGACO consistently exhibited the highest values, approaching 25.1 s at Q = 90, indicating significant computational overhead due to its complex model structure. SRIS-Net had slightly lower encoding times than MGACO, measuring 23.6 s at Q = 90 and 22.5 s at Q = 50, though its overall trend still fluctuated with changes in compression strength. In contrast, ISA-ELCN performed optimally, maintaining encoding times below 23 s across different compression strengths with minimal fluctuations in the curve. This fully demonstrated that the introduction of a lightweight convolutional structure effectively reduced the computational burden of the model during the information embedding process. In Fig 11(b), the trend in decoding time was consistent with that of encoding time. MGACO approached 25.0 s at Q = 90, SRIS-Net was at 23.4 s, while ISA-ELCN remained around 22.3 s. The results indicated that ISA-ELCN demonstrated high stability and efficiency advantages in both encoding and decoding stages, ensuring low computational complexity and reflecting its applicability in handling complex compression conditions in practical applications. The analysis of distortion tolerance and real-time performance of different models under various JPEG compression quality factors is presented in Table 4.

**Table 4:** Distortion tolerance and real-time performance analysis.

JPEG Q	Method	PSNR (dB)	SSIM	Inference time (ms)
30	MGACO	37.5	0.942	23.3
	SRIS-Net	39.1	0.955	19.2
	ISA-ELCN	40.8	0.969	14.7
50	MGACO	37.8	0.945	23.4
	SRIS-Net	39.3	0.957	19.3
	ISA-ELCN	41.2	0.970	14.8
70	MGACO	38.0	0.947	23.5
	SRIS-Net	39.4	0.958	19.4
	ISA-ELCN	41.1	0.971	14.9
90	MGACO	38.1	0.948	23.6
	SRIS-Net	39.5	0.959	19.5
	ISA-ELCN	41.2	0.972	15.1

**Fig 12:**
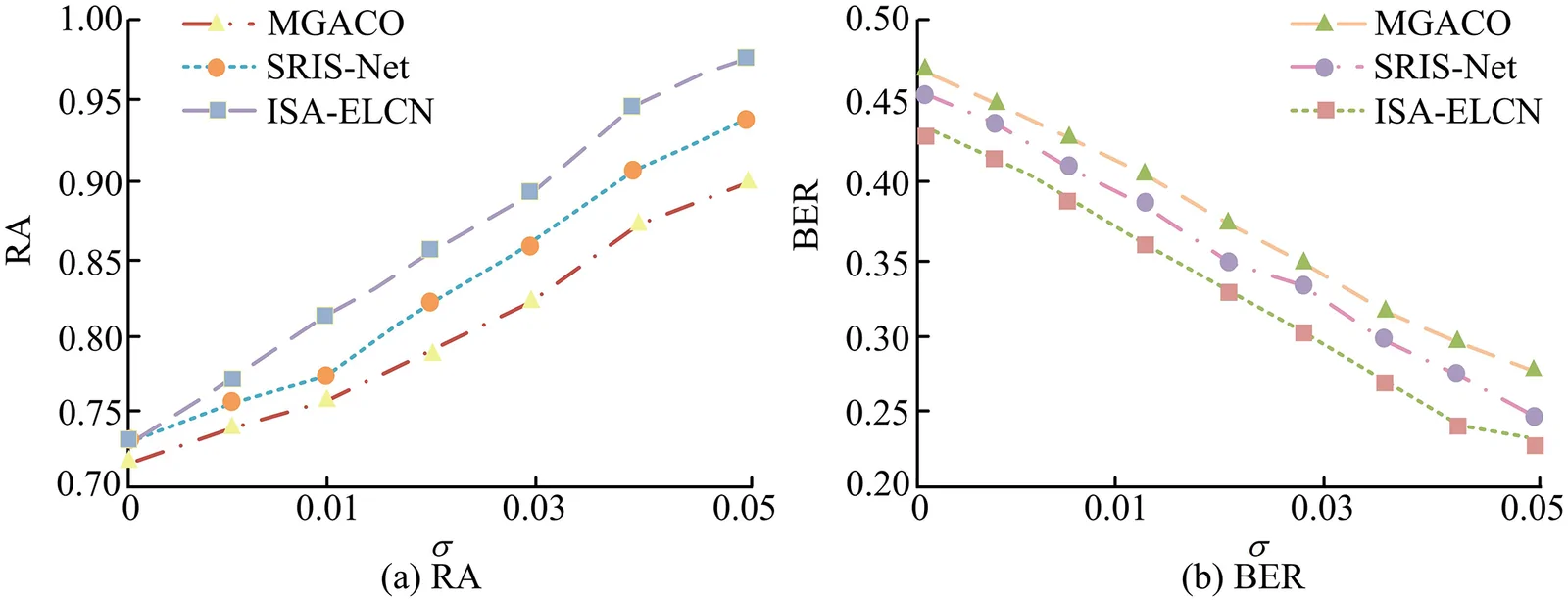
RA and BER under different noise interferences.

As shown in Table 4, In terms of image quality, as the Q value increased from 30 to 90, the PSNR and SSIM of all three methods exhibited a gradual upward trend, indicating that higher compression quality resulted in better fidelity of stego images after steganography. Among them, ISA-ELCN consistently maintained a PSNR above 40 dB, reaching a maximum of 41.2 dB, significantly higher than those of SRIS-Net and MGACO. In terms of SSIM, ISA-ELCN’s results remained stable within the range of 0.969–0.972, which was also significantly better than SRIS-Net’s 0.955–0.959 and MGACO’s 0.942–0.948, demonstrating ISA-ELCN’s ability to preserve high visual quality and structural similarity under stronger compression. Regarding inference efficiency, MGACO’s inference time consistently ranged from 23.3 to 23.6 ms, while SRIS-Net performed better at around 19 ms. In contrast, ISA-ELCN, benefiting from its lightweight convolutional structure and parameter reorganization strategy, required as little as 14.7 ms, maintaining a time of approximately 15 ms across all compression strengths, thus achieving the fastest inference speed and the best stability. From the perspective of network architecture, ISA-ELCN converges faster and shows better synergistic improvement in PSNR and SSIM metrics compared to SRIS Net and MGACO, mainly due to EtENet’s dual branch feature extraction and hierarchical transmission design. On the one hand, EtENet models high-frequency texture feature branches and low-frequency structural feature branches in parallel during the encoding stage, enabling the network to embed fine details in regions without damaging the overall structure, thus simultaneously balancing PSNR and SSIM. On the other hand, introducing a hierarchical feature propagation mechanism similar to DenseNet significantly alleviates the gradient decay problem in deep networks, enabling effective gradients to be transmitted faster to shallow embedding modules, thereby reducing training epochs and improving convergence stability. In addition, by combining the structural reparameterization of RepVGG, the computational graph in the inference stage is simplified while retaining the ability of multi branch convolution expression, making it easier for the model to form an optimized trajectory with simultaneous improvement of PSNR and SSIM during the training stage. To further verify the robustness of the introduced approach in complex channel environments, the study also introduced interference noise of varying intensities into the stego images and compared its performance with that of the comparative methods in regard to RA, BER, and image quality, with the results shown in Fig 12.

Fig 12(a) and 12(b) respectively illustrate the changes in RA and BER for the three methods under noise interference. In Fig 12(a), MGACO consistently exhibited the lowest RA, measuring 0.78 at σ =0.01 and improving to 0.91 at σ =0.05. SRIS-Net performed relatively better, reaching 0.80 at σ =0.01 and approaching 0.94 at σ =0.05. ISA-ELCN, however, maintained the best performance across the entire range of noise intensities, with an RA close to 0.82 at σ =0.01 and further improving to 0.96 at σ =0.05, significantly outperforming the other methods and demonstrating more stable recovery capability under noise interference. The overall trend in Fig 12(b) was opposite to that of RA. At σ =0.01, MGACO’s BER was 0.45, SRIS-Net’s was 0.43, while ISA-ELCN’s was only 0.41. When σ =0.05, MGACO’s BER remained at 0.28, SRIS-Net’s decreased to 0.25, and ISA-ELCN’s further dropped to 0.22, consistently maintaining the lowest level. Thus, under different noise intensity conditions, ISA-ELCN achieved higher RA and lower BERs, showcasing its stronger robustness and practical value in noise-interfered environments. To further verify the robustness of the proposed ISA-ELCN algorithm under complex channel conditions, in addition to JPEG compression, various common image distortion operations were introduced, including scaling (0.75 ×), cropping (90%), and compared with video watermarking methods based on improved gravity search algorithm (VW-MGSA) [32] and facial reconstruction GAN (FRe GAN) method [33]. The results are shown in Table 5.

**Table 5:** Performance comparison of steganography methods in multi distortion scenarios.

Type	Method	BER	PSNR	SSIM
JPEG Q = 30	SRIS-Net	0.54	31.2	0.939
	MGACO	0.49	31.6	0.944
	VW-MGSA	0.46	32.0	0.947
	FRe-GAN	0.44	32.2	0.954
	ISA-ELCN	0.38	32.9	0.956
Resizing 0.75×	SRIS-Net	0.57	30.5	0.931
	MGACO	0.52	30.8	0.936
	VW-MGSA	0.48	31.4	0.942
	FRe-GAN	0.47	31.6	0.944
	ISA-ELCN	0.41	32.1	0.951
Cropping 90%	SRIS-Net	0.62	30.1	0.926
	MGACO	0.55	30.5	0.931
	VW-MGSA	0.52	30.8	0.935
	FRe-GAN	0.51	31.0	0.937
	ISA-ELCN	0.43	31.5	0.947

In Table 5, the proposed ISA-ELCN model achieved the best performance in three typical distortion scenarios. Under the condition of JPEG Q = 30, the BER of ISA-ELCN is 0.38, significantly lower than SRIS Net’s 0.54 and MGACO’s 0.49. Compared with VW-MGSA and FRe GAN, it further reduces the BER, indicating that the model has stronger ability to preserve embedded information when dealing with compression noise, and its PSNR and SSIM reach 32.9 dB and 0.956, respectively, maintaining good structural and visual quality. Under scaling conditions, the BER of the model decreased to 0.41, PSNR reached 32.1 dB, and SSIM was 0.951, still better than other methods, indicating its robustness under geometric scale changes and better structural consistency. In the cropping scenario, although the overall BER has increased, ISA-ELCN still maintains the lowest error rate of 0.43, PSNR at 31.5 dB, and SSIM at 0.947, indicating its ability to effectively recover embedded information in the absence of content and maintain image structural features as much as possible. Overall, ISA-ELCN achieved a balance between the lowest BER and the strongest structural preservation under all three types of distortion conditions, verifying its advantages in steganographic robustness. Its performance improvement has universality across distortion types, not only outstanding in compression scenarios, but also maintaining good stability and structural fidelity under the influence of various disturbances. ISA-ELCN takes into account robustness, interpretability, and lightweight deployment in its design. In terms of robustness, the model utilizes structure preservation and adversarial training to enable stable recovery of steganographic information even under interference such as compression, scaling, and pruning, demonstrating adaptability to complex channel conditions. In terms of interpretability, its encoding extraction dual path and high low frequency joint modeling mechanism make the feature flow clear and traceable, facilitate visualization and analysis of embedding areas, and support security review and policy optimization.

### 3.3 Generalization and statistical significance validation across multiple datasets

To further validate the generalization ability, comparative sufficiency, and statistical reliability of the ISA-ELCN method, ImageNet and COCO subsets were introduced for generalization validation. Simultaneously, the performance of the wavelet transform-based DWT-SVD method, the optimization-strategy-based LSB-GA method, and the DL steganography method SRIS-Net was compared. All experiments were repeated 10 times. Results are expressed as mean ± standard deviation, and paired t-tests were used to compare differences. *p* < 0.05 indicated statistical significance. The results are shown in Table 6.

**Table 6:** Generalization performance comparison of different steganography methods.

Dataset	Method	PSNR (dB)	SSIM	BER	Steganalysis AUC
ImageNet	DWT-SVD	36.94 ± 0.39	0.936 ± 0.006	0.029 ± 0.004	0.641 ± 0.022
	LSB-GA	37.58 ± 0.36	0.941 ± 0.005	0.025 ± 0.003	0.596 ± 0.020
	SRIS-Net	38.42 ± 0.35	0.948 ± 0.005	0.018 ± 0.003	0.472 ± 0.020
	ISA-ELCN	39.86 ± 0.30*	0.962 ± 0.004*	0.011 ± 0.002*	0.421 ± 0.017*
COCO	DWT-SVD	36.61 ± 0.42	0.932 ± 0.007	0.033 ± 0.004	0.658 ± 0.023
	LSB-GA	37.12 ± 0.40	0.938 ± 0.006	0.028 ± 0.004	0.617 ± 0.021
	SRIS-Net	38.15 ± 0.38	0.945 ± 0.006	0.021 ± 0.003	0.486 ± 0.021
	ISA-ELCN	39.52 ± 0.33*	0.958 ± 0.004*	0.013 ± 0.002*	0.435 ± 0.019*

Note: * indicates a statistically significant difference compared to the best comparison method on the same dataset, *p* < 0.05.

Table 6 shows that on the ImageNet subset, ISA-ELCN achieved a PSNR of 39.86 dB, an SSIM of 0.962, a BER reduced to 0.011, and a Steganalysis AUC reduced to 0.421, all outperforming DWT-SVD, LSB-GA, and SRIS-Net. On the COCO subset, despite more complex image scenes and more pronounced texture variations, ISA-ELCN maintained a PSNR of 39.52 dB, an SSIM of 0.958, a BER of 0.013, and a Steganalysis AUC of 0.435. Compared to the best comparison method, ISA-ELCN’s improvements in key metrics were all statistically significant (*p* < 0.05), indicating that the proposed method maintained good image quality, secret information recovery capability, and steganalysis resistance under different image sources. To further verify the lightweight design effectiveness of ISA-ELCN, the model complexity was also evaluated. The results are shown in Table 7.

**Table 7:** Lightweight efficiency comparison of different methods.

Method	Params (M)	FLOPs (G)	Memory (MB)	Inference time (ms)
DWT-SVD	–	–	482	24.30 ± 0.45
LSB-GA	–	–	515	21.80 ± 0.39
SRIS-Net	8.72	15.4	612	19.50 ± 0.34
ISA-ELCN	5.46	9.82	428	15.10 ± 0.28*

Note: “-” indicates that the metric is not applicable. DWT-SVD and LSB-GA are not deep neural network models, so the number of neural network parameters and FLOPs cannot be statistically analyzed. * indicates that the difference compared with the best comparison method is statistically significant, *p* < 0.05.

**Fig 13:**
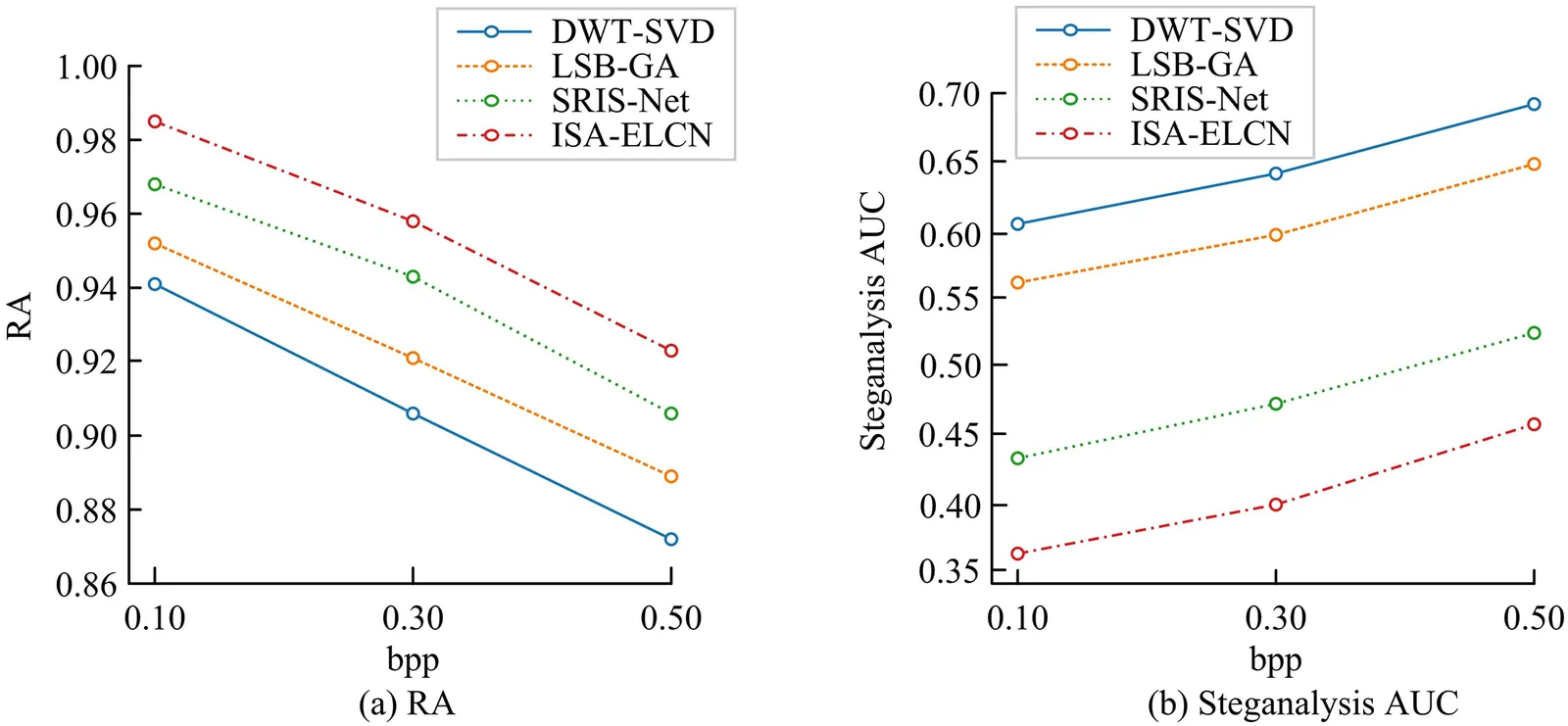
Performance comparison under different bpp conditions.

Table 7 shows that compared with the DL steganography method SRIS-Net, the number of parameters of ISA-ELCN decreased from 8.72 M to 5.46 M, and the number of Floating Point Operations (FLOPs) decreased from 15.40 G to 9.82 G, indicating that the improved convolution module and structural reparameterization mechanism could effectively reduce the computational cost of the model. Meanwhile, ISA-ELCN’s memory usage was 428 MB, lower than DWT-SVD, LSB-GA, and SRIS-Net; its inference time was 15.10 ms, also lower than all the comparison methods. This showed that ISA-ELCN reduced model complexity and inference overhead while maintaining the feature representation capabilities of deep models, making it suitable for image steganography applications in resource-constrained environments. Finally, the study further compared the information recovery ability and anti-detection ability of the four methods under different bpp conditions. The results are shown in Fig 13.

As shown in Fig 13(a), the RA of all methods decreased as the bpp increased from 0.10 to 0.50, indicating that with increased embedding capacity, the carrier image needs to carry more secret information, thus increasing the difficulty of secret information recovery. However, compared to DWT-SVD, LSB-GA, and SRIS-Net, ISA-ELCN consistently maintained the highest RA under all bpp conditions, demonstrating its good information recovery stability under different embedding capacities. Combined with Fig 13(b), the Steganalysis AUC of all methods generally increased with increasing bpp, indicating that higher embedding capacity enhanced image perturbation and steganographic traces, thereby increasing the risk of detection. In contrast, ISA-ELCN consistently maintained the lowest Steganalysis AUC under different bpp conditions, indicating that the proposed method had stronger concealment and anti-detection capabilities. In summary, ISA-ELCN maintained superior information recovery and anti-detection performance even under higher embedding capacity conditions, achieving a good balance between embedding capacity, RA, and concealment.

## 4. Discussion and Conclusion

To address the shortcomings of existing image steganography methods, such as insufficient robustness under complex channel conditions, high model computational overhead, and limited stability of the embedding-extraction process, this paper proposed an image steganography algorithm, ISA-ELCN, based on EtENet and a lightweight convolutional network. Improved Inception-V3 and Xception convolutional blocks were introduced into the encoding network to enhance multi-scale spatial and channel feature extraction capabilities. Simultaneously, the RepVGG reparameterization mechanism was combined to reduce the computational complexity of the inference stage while maintaining feature representation capabilities. In the decoding network, a dense connection mechanism was used to strengthen the cross-layer propagation and reuse of shallow embedded features, thereby reducing the attenuation of secret information during deep convolution propagation. The introduction of a discriminant network further enhanced the distribution consistency between the stegana image and the carrier image, enabling the model to improve the accuracy of secret information recovery while possessing stronger anti-detection capabilities.

Experimental results showed that ISA-ELCN outperformed SRIS-Net and MGACO in terms of image quality, information recovery, and anti-interference capabilities. On the BOSSBase 1.01 dataset, the BER of the complete model decreased to 0.008, and the Steganalysis AUC decreased to 0.398, indicating that the proposed method could reduce the risk of being identified by steganalysis detectors while ensuring the accuracy of secret information recovery. Ablation experiments further showed that the improved convolutional blocks enhanced the sufficiency of feature extraction, dense connections enhanced the deep recovery capability of secret information, and the discriminative network improved the distribution concealment of steganalyst images.

In terms of image quality, ISA-ELCN achieved a PSNR of 40.7 dB, an SSIM of 0.969, and an LPIPS of 0.055 on the test set. This indicated that the proposed method could still maintain the pixel fidelity, structural consistency, and perceptual quality of the carrier image well after embedding secret information. Compared with traditional steganalysis methods based on manual rules or optimization search, ISA-ELCN achieved adaptive adjustment of embedding region and embedding strength through end-to-end learning [7,9,30]. Compared to existing deep steganography methods, lightweight convolutional structures and reparameterization strategies further reduced redundant computation, achieving a better balance between performance and efficiency [5,10,31].

In complex channel experiments, ISA-ELCN demonstrated good compression robustness and noise resistance. With a compression quality factor Q = 30, the method maintained an RA of 0.82 and a BER of 0.38; when Q = 90, the RA increased to 0.98, and the BER decreased to 0.23. Under a noise standard deviation of 0.05, the RA and BER were 0.96 and 0.22, respectively. This indicated that multi-scale feature modeling and cross-layer feature reuse helped improve the recoverability of secret information under compression and noise perturbation. Furthermore, ISA-ELCN exhibited good generalization performance on ImageNet and COCO subsets, and demonstrated higher deployment efficiency in terms of parameter count, FLOPs, memory footprint, and inference time.

While ISA-ELCN achieved a good balance in terms of steganalysis capacity, image quality, robustness, and inference efficiency, it still has certain limitations. First, current experiments primarily validated typical perturbations such as compression, noise, scaling, and cropping, but did not adequately consider real-world combined distortions such as blurring, color shifts, multiple compressions, and complex geometric transformations. Second, although the model reduced inference overhead, it still relied heavily on GPU resources during training, and its deployment on edge devices requires further optimization. Finally, the current method was mainly geared towards grayscale images, and its adaptability to color image channel correlation and multimodal feature alignment needs improvement.

Future research will further optimize the model structure and application scenarios. On the one hand, it will introduce a lightweight Transformer, channel grouping embedding, color adaptive normalization, and dynamic embedding intensity allocation mechanisms to improve modeling capabilities for color images and complex texture scenes. On the other hand, it will combine self-supervised pre-training and perturbation consistency constraints, and extend the model to multi-source datasets, color image steganography, and multimodal information hiding tasks to improve its generalization ability and deployment feasibility in complex channels and edge security applications.
